# Synthesis and crystal structure of 2,9-di­amino-5,6,11,12-tetra­hydro­dibenzo[*a*,*e*]cyclo­octene

**DOI:** 10.1107/S2056989024004468

**Published:** 2024-05-21

**Authors:** Nichole Valdez, Eric Nagel, Erica Redline, Mark Rodriguez, Chad Staiger, Jason Dugger, Jeffrey Foster

**Affiliations:** a Sandia National Laboratories, 1515 Eubank Blvd. SE, Albuquerque, NM 87123, USA; b Oak Ridge National Laboratory, 5200 1 Bethel Valley Rd., Oak Ridge, Tennessee 37831, USA; Katholieke Universiteit Leuven, Belgium

**Keywords:** crystal structure, thermoset polymer, polymers, CTE, characterization

## Abstract

The *cis*- form of di­amino­dibenzo­cyclo­octane (DADBCO, C_16_H_18_N_2_) is of inter­est as a negative coefficient of thermal expansion (CTE) material. The crystal structure was determined through single-crystal X-ray diffraction at 100 K and is presented herein.

## Abstract 2

Successful separation of *cis*- and *trans*-di­amino­dibenzo­cyclo­octane (DADBCO) allows the crystal structure solution of *cis*-DADBCO.

## 1. Chemical context

Thermoset polymers are high performance materials that demonstrate excellent chemical, thermal, and mechanical stability at low weight and cost, making them ubiquitous in a wide range of applications such as insulating layers, encapsulants, adhesives, barriers, and composites (Biron, 2013 ▸; Brostow *et al.*, 2014 ▸; Dickie *et al.*, 1988 ▸; Pascault *et al.*, 2002 ▸; Guo, 2018 ▸). Because these applications involve an inter­face between different materials, the thermal expansion behaviors of each constituent must be considered to achieve suitable performance. Most solid materials exhibit a positive coefficient of thermal expansion (CTE), the rate at which thermal expansion occurs during positive temperature change. Large differences in CTE between the various materials in composites and devices results in inter­nal thermomechanical stress at inter­faces, which in turn reduces service life and may initiate device failure (Okura *et al.*, 2000 ▸; de Vreugd *et al.*, 2010 ▸). 
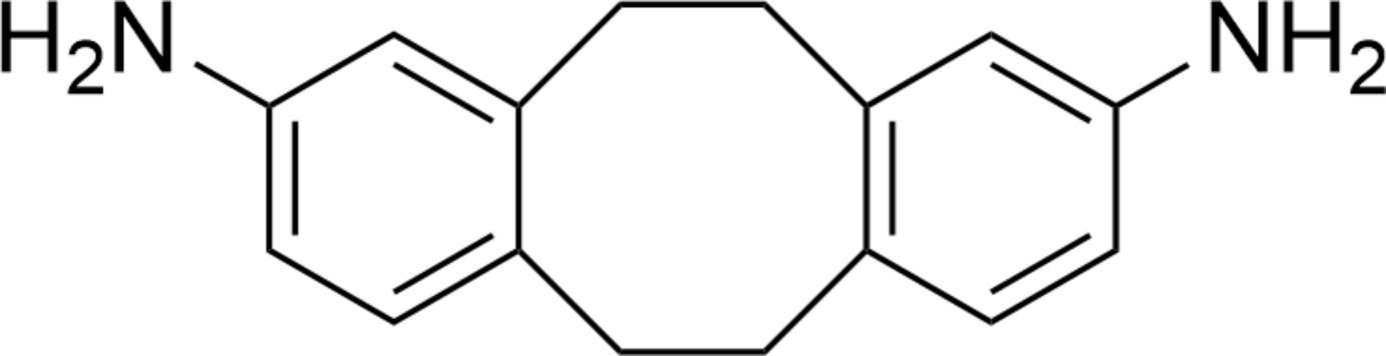


One strategy to mitigate CTE incompatibilities is the covalent incorporation of thermally activated contractile units into the polymer (Shen *et al.*, 2013 ▸). These units counteract the thermal expansion during heating, reducing the CTE below that of the parent material. Materials capable of zero or even negative CTE are achievable with this method. Dibenzo­cyclo­octane (DBCO) is one such unit, which achieves a thermally activated volume decrease by undergoing a reversible twist-boat to chair conformational change (Shen *et al.*, 2013 ▸; Wang *et al.*, 2018 ▸; Fu *et al.*, 2020 ▸). Di­amino­dibenzo­cyclo­octane (DADBCO), an aminated derivative, is also able to undergo this CTE modifying conformational change. However, it was found that ep­oxy resins incorporating 2,2′-DADBCO (*cis*) demonstrated negative CTE behavior while those utilizing 2,3′-DADBCO (*trans*) did not (Foster *et al.*, 2021 ▸). These two materials are not differentiable by most characterization methods including IR, MS, and NMR. In addition, melting points are unreliable due to the difficulty of separation of these two isomers.

## 2. Structural Commentary

**Figure 1:**
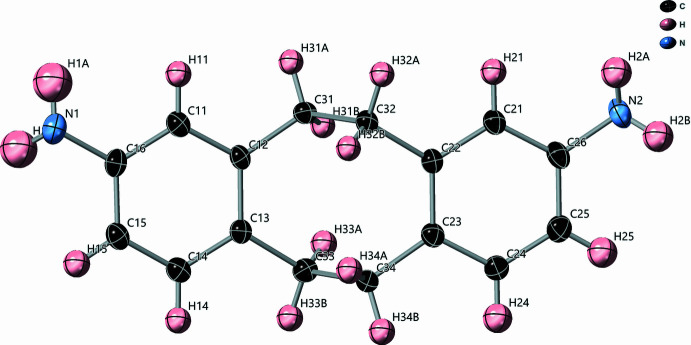
Displacement ellipsoid plot of *cis*-DADBCO with atom labels. Ellipsoids are drawn at the 50% probability level.

The *cis*-DADBCO mol­ecules (point group *C*2) crystallizes in the chair conformation in space group *Pna*2_1_. The structure was determined at 100 K and is illustrated in Fig. 1 ▸. The carbon rings are labeled as Ring 1: C11–C16 with nitro­gen N1 connected to Ring 1 at C16, Ring 2: C21–C26 with N2 connected to Ring 2 at C26. The center cyclo­octane contains carbon atoms: C12, C13, C31–C34, C22, C23. The plane of Ring 1 (C11–C16) makes a 59.9 (1)° angle with the plane that contains the four central atoms of the chair cyclo­octane, C31–34. Nitro­gen N1 is essentially planar with Ring 1 with a deviation from the plane of −0.025 (4) Å. The plane of Ring 2 (C21–26) makes a 56.7 (1)° angle with the aforementioned cyclo­octane plane. Nitro­gen N2 is essentially planar with Ring 2 with a deviation from the plane of 0.026 (4)°. The puckering parameters (Cremer & Pople, 1975 ▸) of the center cyclo­octane are: total puckering amplitude *Q* = 0.906 (4) Å, *q*_2_ = 0.024 (4) Å φ_2_ = 146 (9)°, *q*_3_ = 0.906 (4) Å φ_3_ = 111.6 (2)°, q_4_ = −0.005 (4) Å.

## 3. Supra­molecular Features

**Figure 2:**
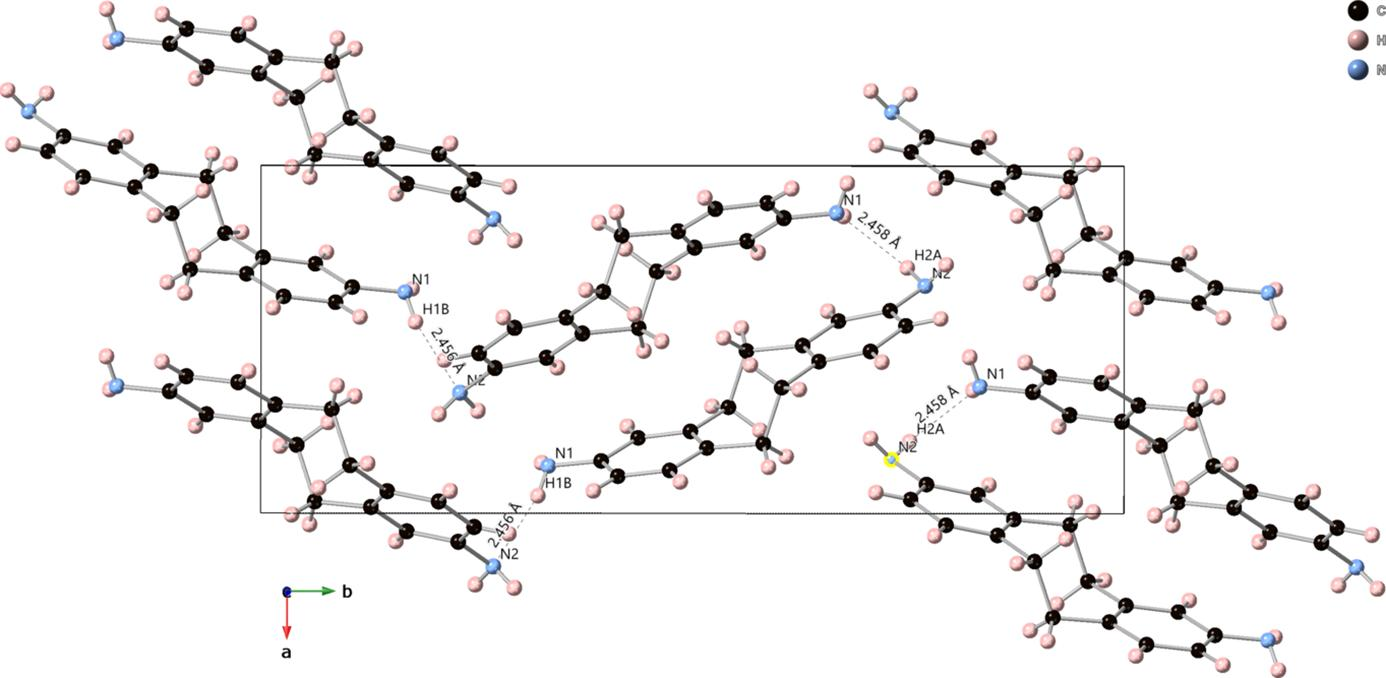
Hydrogen bonding in the crystal packing of the title compound. Incomplete mol­ecules in the range are omitted for clarity. Displacement ellipsoids are drawn at 50% probability.

Hydrogen bonding appears to be possible between the two amine groups from one independent mol­ecule to the next through hydrogen H1*B* on nitro­gen N2 to nitro­gen N1 (bond length 2.452 Å) and through hydrogen H2*A* on nitro­gen N2 to nitro­gen N1 (bond length 2.458 Å). This information is summarized in Table 1 ▸ and illustrated in Fig. 2 ▸. N—H..π and C—H..π inter­actions are also included in Table 1 ▸.

## 4. Database survey

A search was performed on the Cambridge Structural Database (CSD Version 5.45, March 2024; Groom *et al.*, 2016 ▸) using ConQuest (Version 2024.1.0), and this aminated structure was not found. At time of submission there were eight 6,8,6 ring system entries in the CSD, with cyclo­octane as the central ring, no additional rings linked, and no chemical substitutions in the core 6,8,6 carbon rings. A search of the chemical formula also yielded no results. An overview of 6,8,6 ring systems can be found in Domiano *et al.* (1992 ▸). The 6,8,6 motif also appears in circulene systems, see Miyoshi *et al.* (2022 ▸).

## 5. Synthesis and crystallization

**Synthesis of dibenzo­cyclo­octane (DBCO)**

The synthesis of DBCO was carried out according to a literature procedure (Franck *et al.*, 2012 ▸). An oven-dried three-necked round-bottom flask equipped with a stir bar was charged with lithium metal (3.30 g, 478 mmol, 2.5 equiv) and 100 mL of anhydrous THF in an Ar-filled glovebox. The flask was sealed with two rubber septa and vacuum adapter with a stopcock. The flask was removed from the glovebox and connected to a Schlenk line. The flask was fitted with a reflux condenser and pressure-equalizing addition funnel under N_2_ flow. A solution of α,α′-di­bromo-*o*-xylene (50.2 g, 190 mmol, 1 equiv) in 100 mL of anhydrous THF was prepared and transferred to the addition funnel. This solution was added dropwise to the Li suspension with vigorous stirring under an N_2_ atmosphere. After ∼1/4 of this solution had been added, the reaction mixture began to reflux, and the addition was paused to allow the exotherm to subside. After 5 min, the dropwise addition was resumed, taking 1 h to add the remaining solution. The addition funnel was replaced with a glass stopper and the reaction mixture was heated at reflux overnight under N_2_. Complete consumption of the starting material was confirmed by TLC in petroleum ether. The reaction flask was cooled in an ice bath and the reaction mixture was then carefully filtered over a glass frit to remove unreacted Li. The filtrate was concentrated *in vacuo* and was re-suspended in 200 mL of CH_2_Cl_2_. The resulting suspension was filtered over a pad of silica gel and the silica gel pad was washed with an additional 200 mL of CH_2_Cl_2_. The combined filtrates were dried over Na_2_SO_4_ and concentrated *in vacuo*, yielding 20.7 g of viscous yellow oil that solidified upon standing. The crude product was further purified *via* Kugelrohr distillation at 463 K under vacuum to afford the pure product as a white crystalline solid.

**Synthesis of di­nitro dibenzo­cyclo­octane (DNDBCO)**

DBCO (4.55 g, 21.9 mmol, 1 equiv) was dissolved in 200 mL of CH_2_Cl_2_ in a round-bottom flask equipped with a stir bar. The flask was fitted with a pressure-equalizing addition funnel and was placed in an ice bath. To the flask was added 25 mL HNO_3_ (∼20 equiv) dropwise *via* the addition funnel at 273 K. During the addition, the reaction mixture developed a deep red color. The flask was removed from the ice bath and allowed to warm to room temperature. The reaction mixture was stirred at room temperature for 2 h, during which time the color of the reaction mixture changed from deep red to yellow–orange. The reaction mixture was poured into a beaker containing 300 mL of cold deionized (DI) H_2_O to quench the reaction, and this biphasic mixture was transferred to a separatory funnel. The aqueous layer was discarded, and the organic layer was washed subsequently with DI H_2_O, saturated NaHCO_3_ solution (2×), and brine, dried over Na_2_SO_4_, filtered, and concentrated *in vacuo*. Upon further drying under vacuum, a yellow solid was obtained that was used in the next step without further purification.

**Synthesis of di­amino dibenzo­cyclo­octane (DADBCO)**

A three-necked round-bottom flask equipped with a stir bar was charged with 10% Pd/C (0.89 g, 5 mol% Pd relative to DNDBCO). The flask was fitted with two rubber septa and a vacuum adapter and was placed under an N_2_ atmosphere. To the flask was added 150 mL of MeOH followed by DNDBCO (5.05 g, 16.9 mmol, 1 equiv) under N_2_ flow. The rubber septa were replaced with vacuum adapters attached to H_2_-filled balloons, and the N_2_ atmosphere was exchanged for H_2_ *via* five vacuum/H_2_-back-fill cycles. The reaction mixture was then stirred overnight at room temperature under H_2_, over which time the solid DNDBCO slowly dissolved. The complete consumption of the starting material was confirmed by TLC in 2:1 hexa­ne/ethyl acetate. The reaction mixture was filtered over a pad of celite to remove the Pd/C and the filtrate was concentrated *in vacuo*. The resulting dark-red oil was re-dissolved in 1*M* HCl solution and was washed with CH_2_Cl_2_ (3×). The pH of the solution was then adjusted to pH >10 using ∼4*M* NaOH solution, and the precipitated product was extracted with EtOAc (2×). The combined EtOAc solutions were washed with DI H_2_O and brine, dried over Na_2_SO_4_, filtered, and concentrated *in vacuo*. Further drying under vacuum afforded the product as a light-pink solid (3.05 g, 76% yield). Note: DADBCO was obtained as a mixture of isomers, where the ratio of *ortho*/*meta* anilines was ∼2:3. The various isomers could be isolated *via* exhaustive silica gel chromatography, eluting with a gradient from 0–50% EtOAc in hexane. In particular, the 2,2′ and 2,3′ isomers were suspected as the last and second to last compounds that eluted from the column, respectively. A suitable crystal of the 2,2′ material was grown for XRD analysis by allowing a saturated hot toluene solution to cool to room temperature over several hours followed by further cooling to 273 K.

## 6. Refinement

Crystal data, data collection, and structure refinement details are summarized in Table 2 ▸. The hydrogen atoms on N2 were placed with a riding-bond model, whereas the hydrogen atoms on N1 were placed manually to match observed electron density. The distance of the manually placed atoms was constrained with DFIX and for the hydrogen atoms on N1 the isotropic thermal parameters were refined without constraints. All other H atoms were generated *via* the riding-bond model and refined with *U*(H) = 1.2*U*_eq_(C/N). The absolute structure was not determined due to the absence of heavy atoms (Flack parameter = 0.3), and the inversion twin law was used for refinement.

## Supplementary Material

Crystal structure: contains datablock(s) I. DOI: 10.1107/S2056989024004468/vm2301sup1.cif

Structure factors: contains datablock(s) I. DOI: 10.1107/S2056989024004468/vm2301Isup2.hkl

PLATON outputs and SHELX plane calculations. DOI: 10.1107/S2056989024004468/vm2301sup3.docx

Supporting information file. DOI: 10.1107/S2056989024004468/vm2301Isup4.cml

CCDC reference: 2355133

Additional supporting information:  crystallographic information; 3D view; checkCIF report

## Figures and Tables

**Table 1:** Hydrogen-bond geometry (Å, °) *Cg*1 and *Cg*2 are the centroids of rings C11–C16 and C21–C26, respectively.

*D*—H⋯*A*	*D*—H	H⋯*A*	*D*⋯*A*	*D*—H⋯*A*
N1—H1*B*⋯N2^i^	0.86 (4)	2.46 (4)	3.295 (5)	165 (4)
N2—H2*A*⋯N1^ii^	0.88	2.46	3.295 (5)	159
N2—H2*B*⋯*Cg*2^iii^	0.88	2.96	3.742 (4)	149
C14—H14⋯*Cg*1^iv^	0.95	2.84	3.572 (4)	135
C32—H32*A*⋯*Cg*1^ii^	0.99	2.78	3.640 (4)	145

Symmetry codes: (i) 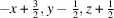 ; (ii) 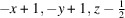 ; (iii) 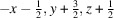 ; (iv) 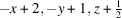 .

**Table 2:** Experimental details

Crystal data	
Chemical formula	C_16_H_18_N_2_
*M*_r_	238.32
Crystal system, space group	Orthorhombic, *P* *n* *a*2_1_
Temperature (K)	100
*a*, *b*, *c* (Å)	8.8641 (3), 22.0075 (6), 6.2771 (2)
*V* (Å^3^)	1224.52 (7)
*Z*	4
Radiation type	Cu *K*α
μ (mm^−1^)	0.59
Crystal size (mm)	0.1 × 0.1 × 0.01

Data collection	
Diffractometer	Bruker APEXII CCD
Absorption correction	Multi-scan (*SADABS*; Krause *et al.*, 2015 ▸)
*T*_min_, *T*_max_	0.459, 0.754
No. of measured, independent and observed [*I* > 2σ(*I*)] reflections	39813, 2060, 1781
*R*_int_	0.118
(sin θ/λ)_max_ (Å^−1^)	0.588

Refinement	
*R*[*F* ^2^ > 2σ(*F* ^2^)], *wR*(*F* ^2^), *S*	0.046, 0.106, 1.08
No. of reflections	2060
No. of parameters	173
No. of restraints	172
H-atom treatment	H atoms treated by a mixture of independent and constrained refinement
Δρ_max_, Δρ_min_ (e Å^−3^)	0.18, −0.22
Absolute structure	Refined as an inversion twin
Absolute structure parameter	0.3 (12)

Computer programs: *APEX2* and *SAINT* (Bruker, 2019 ▸), *SHELXT* (Sheldrick, 2015*a* ▸), *SHELXL* (Sheldrick, 2015*b* ▸), and *OLEX2* (Dolomanov *et al.*, 2009 ▸).

## supplementary crystallographic information

### Crystal data

**Table:**

C_16_H_18_N_2_	*D*_x_ = 1.293 Mg m^−^^3^
*M**_r_* = 238.32	Cu *K*α radiation, λ = 1.54178 Å
Orthorhombic, *P**n**a*2_1_	Cell parameters from 8510 reflections
*a* = 8.8641 (3) Å	θ = 4.0–73.6°
*b* = 22.0075 (6) Å	µ = 0.59 mm^−^^1^
*c* = 6.2771 (2) Å	*T* = 100 K
*V* = 1224.52 (7) Å^3^	Needle, clear light green
*Z* = 4	0.1 × 0.1 × 0.01 mm
*F*(000) = 512	

### Data collection

**Table:**

Bruker APEXII CCD diffractometer	1781 reflections with *I* > 2σ(*I*)
φ and ω scans	*R*_int_ = 0.118
Absorption correction: multi-scan (SADABS; Krause *et al.*, 2015)	θ_max_ = 65.0°, θ_min_ = 4.0°
*T*_min_ = 0.459, *T*_max_ = 0.754	*h* = −10→10
39813 measured reflections	*k* = −25→25
2060 independent reflections	*l* = −6→7

### Refinement

**Table:**

Refinement on *F*^2^	Hydrogen site location: mixed
Least-squares matrix: full	H atoms treated by a mixture of independent and constrained refinement
*R*[*F*^2^ > 2σ(*F*^2^)] = 0.046	*w* = 1/[σ^2^(*F*_o_^2^) + (0.0353*P*)^2^ + 0.9951*P*] where *P* = (*F*_o_^2^ + 2*F*_c_^2^)/3
*wR*(*F*^2^) = 0.106	(Δ/σ)_max_ < 0.001
*S* = 1.08	Δρ_max_ = 0.18 e Å^−^^3^
2060 reflections	Δρ_min_ = −0.22 e Å^−^^3^
173 parameters	Absolute structure: Refined as an inversion twin
172 restraints	Absolute structure parameter: 0.3 (12)
Primary atom site location: shelXT	

### Special details

**Table:**

Geometry. All esds (except the esd in the dihedral angle between two l.s. planes) are estimated using the full covariance matrix. The cell esds are taken into account individually in the estimation of esds in distances, angles and torsion angles; correlations between esds in cell parameters are only used when they are defined by crystal symmetry. An approximate (isotropic) treatment of cell esds is used for estimating esds involving l.s. planes.
Refinement. Refined as a 2-component inversion twin.

### Fractional atomic coordinates and isotropic or equivalent isotropic displacement parameters (Å^2^)

**Table:**

	*x*	*y*	*z*	*U*_iso_*/*U*_eq_	
N1	0.8644 (4)	0.33304 (16)	0.4674 (6)	0.0276 (8)	
H1A	0.855 (5)	0.325 (2)	0.332 (4)	0.058 (18)*	
H1B	0.950 (4)	0.321 (2)	0.516 (7)	0.052 (16)*	
N2	0.3459 (4)	0.76938 (14)	0.2091 (6)	0.0295 (9)	
H2A	0.293746	0.748604	0.114219	0.035*	
H2B	0.284518	0.793724	0.279538	0.035*	
C11	0.7832 (4)	0.43707 (17)	0.3906 (6)	0.0205 (9)	
H11	0.750058	0.424326	0.253757	0.025*	
C12	0.7645 (4)	0.49766 (16)	0.4496 (6)	0.0181 (8)	
C13	0.8118 (4)	0.51680 (17)	0.6518 (6)	0.0186 (8)	
C14	0.8763 (4)	0.47369 (16)	0.7896 (7)	0.0211 (8)	
H14	0.908901	0.485974	0.927203	0.025*	
C15	0.8932 (4)	0.41367 (17)	0.7284 (7)	0.0228 (9)	
H15	0.935284	0.385279	0.825644	0.027*	
C16	0.8497 (4)	0.39431 (17)	0.5276 (7)	0.0217 (9)	
C31	0.6953 (4)	0.54170 (17)	0.2940 (6)	0.0195 (8)	
H31A	0.692418	0.522457	0.151541	0.023*	
H31B	0.760925	0.577985	0.284019	0.023*	
C32	0.5330 (4)	0.56256 (16)	0.3539 (6)	0.0192 (8)	
H32A	0.467057	0.556422	0.228475	0.023*	
H32B	0.495208	0.535789	0.468896	0.023*	
C33	0.7993 (4)	0.58216 (16)	0.7233 (7)	0.0214 (8)	
H33A	0.836432	0.608605	0.606980	0.026*	
H33B	0.866689	0.588157	0.847236	0.026*	
C34	0.6377 (4)	0.60340 (17)	0.7861 (6)	0.0217 (8)	
H34A	0.572848	0.567063	0.802213	0.026*	
H34B	0.642990	0.623702	0.926614	0.026*	
C21	0.4468 (4)	0.66922 (16)	0.2906 (7)	0.0205 (8)	
H21	0.418445	0.656557	0.151499	0.025*	
C22	0.5171 (4)	0.62758 (17)	0.4262 (6)	0.0182 (8)	
C23	0.5629 (4)	0.64650 (17)	0.6289 (6)	0.0213 (8)	
C24	0.5334 (5)	0.70611 (17)	0.6907 (7)	0.0269 (9)	
H24	0.564002	0.719258	0.828207	0.032*	
C25	0.4600 (5)	0.74720 (19)	0.5557 (7)	0.0287 (10)	
H25	0.439745	0.787438	0.602401	0.034*	
C26	0.4170 (4)	0.72879 (17)	0.3533 (7)	0.0235 (9)	

### Atomic displacement parameters (Å^2^)

**Table:**

	*U*^11^	*U*^22^	*U*^33^	*U*^12^	*U*^13^	*U*^23^
N1	0.0259 (19)	0.0224 (18)	0.034 (2)	0.0023 (15)	0.0022 (17)	−0.0011 (16)
N2	0.0270 (18)	0.0243 (18)	0.037 (2)	0.0035 (14)	−0.0119 (16)	0.0037 (15)
C11	0.0109 (18)	0.026 (2)	0.025 (2)	−0.0010 (15)	−0.0004 (15)	0.0018 (16)
C12	0.0108 (17)	0.0217 (19)	0.022 (2)	0.0020 (15)	0.0000 (16)	0.0021 (16)
C13	0.0106 (17)	0.0234 (18)	0.022 (2)	0.0013 (15)	0.0018 (16)	−0.0004 (16)
C14	0.0129 (17)	0.027 (2)	0.023 (2)	−0.0011 (15)	0.0004 (16)	0.0019 (17)
C15	0.0153 (18)	0.027 (2)	0.026 (2)	0.0022 (16)	0.0008 (17)	0.0042 (18)
C16	0.0179 (19)	0.0185 (19)	0.029 (2)	0.0003 (15)	0.0041 (17)	0.0010 (17)
C31	0.0152 (18)	0.0242 (19)	0.019 (2)	0.0001 (15)	−0.0003 (16)	−0.0019 (16)
C32	0.0140 (17)	0.0197 (19)	0.024 (2)	−0.0009 (14)	−0.0024 (16)	−0.0010 (15)
C33	0.0175 (18)	0.0240 (19)	0.023 (2)	−0.0017 (15)	0.0000 (17)	−0.0019 (16)
C34	0.0232 (19)	0.0210 (18)	0.021 (2)	0.0029 (16)	−0.0043 (17)	−0.0023 (16)
C21	0.0157 (18)	0.0207 (18)	0.025 (2)	−0.0007 (15)	−0.0031 (17)	0.0004 (16)
C22	0.0141 (18)	0.0189 (19)	0.022 (2)	−0.0010 (14)	0.0011 (16)	0.0018 (15)
C23	0.0186 (19)	0.0215 (19)	0.024 (2)	0.0000 (15)	−0.0030 (17)	−0.0006 (16)
C24	0.031 (2)	0.023 (2)	0.027 (2)	0.0030 (18)	−0.0071 (17)	−0.0039 (18)
C25	0.032 (2)	0.022 (2)	0.032 (3)	0.0022 (18)	−0.0055 (19)	−0.0045 (18)
C26	0.0185 (19)	0.0217 (19)	0.030 (2)	−0.0010 (16)	−0.0073 (17)	0.0034 (17)

### Geometric parameters (Å, º)

**Table:**

N1—H1A	0.87 (2)	C31—C32	1.556 (5)
N1—H1B	0.86 (2)	C32—H32A	0.9900
N1—C16	1.406 (5)	C32—H32B	0.9900
N2—H2A	0.8817	C32—C22	1.508 (5)
N2—H2B	0.8824	C33—H33A	0.9900
N2—C26	1.419 (5)	C33—H33B	0.9900
C11—H11	0.9500	C33—C34	1.557 (5)
C11—C12	1.394 (5)	C34—H34A	0.9900
C11—C16	1.404 (5)	C34—H34B	0.9900
C12—C13	1.401 (5)	C34—C23	1.520 (5)
C12—C31	1.506 (5)	C21—H21	0.9500
C13—C14	1.406 (5)	C21—C22	1.398 (5)
C13—C33	1.511 (5)	C21—C26	1.394 (5)
C14—H14	0.9500	C22—C23	1.399 (6)
C14—C15	1.384 (5)	C23—C24	1.393 (5)
C15—H15	0.9500	C24—H24	0.9500
C15—C16	1.385 (6)	C24—C25	1.400 (6)
C31—H31A	0.9900	C25—H25	0.9500
C31—H31B	0.9900	C25—C26	1.387 (6)
			
H1A—N1—H1B	111 (4)	C22—C32—C31	116.1 (3)
C16—N1—H1A	116 (3)	C22—C32—H32A	108.3
C16—N1—H1B	107 (3)	C22—C32—H32B	108.3
H2A—N2—H2B	109.3	C13—C33—H33A	108.4
C26—N2—H2A	109.7	C13—C33—H33B	108.4
C26—N2—H2B	109.6	C13—C33—C34	115.4 (3)
C12—C11—H11	119.1	H33A—C33—H33B	107.5
C12—C11—C16	121.9 (4)	C34—C33—H33A	108.4
C16—C11—H11	119.1	C34—C33—H33B	108.4
C11—C12—C13	119.5 (4)	C33—C34—H34A	108.5
C11—C12—C31	119.5 (4)	C33—C34—H34B	108.5
C13—C12—C31	121.0 (3)	H34A—C34—H34B	107.5
C12—C13—C14	118.4 (4)	C23—C34—C33	115.1 (3)
C12—C13—C33	122.2 (3)	C23—C34—H34A	108.5
C14—C13—C33	119.3 (3)	C23—C34—H34B	108.5
C13—C14—H14	119.4	C22—C21—H21	119.0
C15—C14—C13	121.1 (4)	C26—C21—H21	119.0
C15—C14—H14	119.4	C26—C21—C22	121.9 (4)
C14—C15—H15	119.5	C21—C22—C32	118.7 (3)
C14—C15—C16	121.1 (4)	C21—C22—C23	119.2 (3)
C16—C15—H15	119.5	C23—C22—C32	121.9 (3)
C11—C16—N1	121.1 (4)	C22—C23—C34	122.1 (3)
C15—C16—N1	120.9 (4)	C24—C23—C34	119.3 (4)
C15—C16—C11	117.9 (3)	C24—C23—C22	118.6 (4)
C12—C31—H31A	108.7	C23—C24—H24	119.1
C12—C31—H31B	108.7	C23—C24—C25	121.8 (4)
C12—C31—C32	114.2 (3)	C25—C24—H24	119.1
H31A—C31—H31B	107.6	C24—C25—H25	120.2
C32—C31—H31A	108.7	C26—C25—C24	119.6 (4)
C32—C31—H31B	108.7	C26—C25—H25	120.2
C31—C32—H32A	108.3	C21—C26—N2	119.7 (4)
C31—C32—H32B	108.3	C25—C26—N2	121.5 (4)
H32A—C32—H32B	107.4	C25—C26—C21	118.8 (4)
			
C11—C12—C13—C14	−0.3 (5)	C31—C32—C22—C21	108.3 (4)
C11—C12—C13—C33	178.1 (3)	C31—C32—C22—C23	−75.3 (4)
C11—C12—C31—C32	108.7 (4)	C32—C22—C23—C34	2.6 (5)
C12—C11—C16—N1	178.4 (3)	C32—C22—C23—C24	−174.8 (4)
C12—C11—C16—C15	1.8 (5)	C33—C13—C14—C15	−178.4 (3)
C12—C13—C14—C15	0.0 (5)	C33—C34—C23—C22	68.8 (5)
C12—C13—C33—C34	76.1 (5)	C33—C34—C23—C24	−113.8 (4)
C12—C31—C32—C22	109.5 (4)	C34—C23—C24—C25	−177.7 (4)
C13—C12—C31—C32	−71.9 (4)	C21—C22—C23—C34	179.0 (3)
C13—C14—C15—C16	1.3 (5)	C21—C22—C23—C24	1.6 (5)
C13—C33—C34—C23	−107.9 (4)	C22—C21—C26—N2	179.7 (3)
C14—C13—C33—C34	−105.6 (4)	C22—C21—C26—C25	0.7 (6)
C14—C15—C16—N1	−178.8 (3)	C22—C23—C24—C25	−0.2 (6)
C14—C15—C16—C11	−2.1 (5)	C23—C24—C25—C26	−1.0 (6)
C16—C11—C12—C13	−0.6 (5)	C24—C25—C26—N2	−178.3 (4)
C16—C11—C12—C31	178.8 (3)	C24—C25—C26—C21	0.7 (6)
C31—C12—C13—C14	−179.7 (3)	C26—C21—C22—C32	174.7 (3)
C31—C12—C13—C33	−1.3 (5)	C26—C21—C22—C23	−1.9 (5)

### Hydrogen-bond geometry (Å, º)

*Cg*1 and *Cg*2 are the centroids of rings C11–C16 and C21–C26, respectively.

**Table:**

*D*—H···*A*	*D*—H	H···*A*	*D*···*A*	*D*—H···*A*
N1—H1*B*···N2^i^	0.86 (4)	2.46 (4)	3.295 (5)	165 (4)
N2—H2*A*···N1^ii^	0.88	2.46	3.295 (5)	159
N2—H2*B*···*Cg*2^iii^	0.88	2.96	3.742 (4)	149
C14—H14···*Cg*1^iv^	0.95	2.84	3.572 (4)	135
C32—H32*A*···*Cg*1^ii^	0.99	2.78	3.640 (4)	145

Symmetry codes: (i) −*x*+3/2, *y*−1/2, *z*+1/2; (ii) −*x*+1, −*y*+1, *z*−1/2; (iii) −*x*−1/2, *y*+3/2, *z*+1/2; (iv) −*x*+2, −*y*+1, *z*+1/2.
