# Supplementary material for: Synthesis and crystal structure of 2,9-di­amino-5,6,11,12-tetra­hydro­dibenzo[a,e]cyclo­octene
Source: Acta Crystallogr E Crystallogr Commun. 2024 May 21;80(Pt 6):645–8. doi: 10.1107/S2056989024004468 (PMC11151320; doi:10.1107/S2056989024004468)
Supplement: Supplementary file 3 [file e-80-00645-sup3.docx]

PLATON Pi

Analysis of X-H...Cg(Pi-Ring) Interactions (H..Cg < 3.0 Ang. - Gamma < 40.0

Deg)

==============================================================================

- Cg(J) = Center of gravity of ring J (Plane number above)

- H-Perp = Perpendicular distance of H to ring plane J

- Gamma = Angle between Cg-H vector and ring J normal

- X-H..Cg = X-H-Cg angle (degrees)

- X..Cg = Distance of X to Cg (Angstrom)

- X-H, Pi = Angle of the X-H bond with the Pi-plane (i.e. Perpendicular = 90

degrees, Parallel = 0 degrees)

X--H(I) Res(I) Cg(J) [ ARU(J)] H..Cg Transformed J-Plane P,

Q, R, S H-Perp Gamma X-H..Cg X..Cg X-H,Pi

N2 -H2B [ 1] -> Cg2 [ 4465.01] 2.96 0.8870-0.2840-0.3641

-6.2828 2.92 9.61 149 3.742(4) 64

C14 -H14 [ 1] -> Cg1 [ 2765.01] 2.84 -0.9054-0.2162-0.3654

-14.4817 2.75 14.57 135 3.572(4) 42

C32 -H32A [ 1] -> Cg1 [ 2664.01] 2.78 -0.9054-0.2162-0.3654

-4.1626 -2.76 7.93 145 3.640(4) 59

----------

----------------------------------------------

Min or Max 2.780

-2.758 7.9 149.00 3.572 64.00

[ 4465] = -1/2+X,3/2-Y,Z

[ 2765] = 2-X,1-Y,1/2+Z

[ 2664] = 1-X,1-Y,-1/2+Z

The Cg(I) refer to the Ring Centre-of-Gravity numbers given in () in the

Ring-Analysis above

Cg(I) x y z Xo

Yo Zo

Cg1 0.82978(16) 0.45553(7) 0.5896(3) 7.3553(14)

10.0252(15) 3.7010(17)

Cg2 0.48953(18) 0.68757(7) 0.4909(3) 4.3393(16)

15.1316(15) 3.0814(17)

PLATON pucker

==================================================================================

8-Membered Ring ( 3) C12 --> C13 --> C33 --> C34 -->

C23 --> C22 --> C32 --> C31 -->

-----------------------------------------------------------------------------------

sp2 sp2 sp3 sp3

sp2 sp2 sp3 sp3

Dev (A) from CPplane -0.179(4) -0.185(4) 0.429(4) -0.407(4)

0.155(4) 0.175(4) -0.413(4) 0.424(4)

Cs(I)-Asym-Par (Deg) 140.1(3) 137.0(3) 53.7(3) 61.2(3)

140.1(3) 137.0(3) 53.7(3) 61.2(3)

C2(I)-Asym-Par (Deg) 53.8(3) 61.3(3) 140.1(3) 137.0(3)

53.8(3) 61.3(3) 140.1(3) 137.0(3)

Ring Bond Angle(Deg) 121.0(3) 122.2(3) 115.4(3) 115.1(3)

122.1(3) 121.9(3) 116.1(3) 114.2(3)

Tors(I-J) (Deg) -1.3(5) 76.1(5) -107.9(4) 68.8(4)

2.6(5) -75.3(5) 109.5(4) -72.0(4)

Cs(I-J)-Asym-Par (Deg) 4.5(4) 90.4(4) 119.3(4) 87.2(4)

4.5(4) 90.4(4) 119.3(4) 87.2(4)

C2(I-J)-Asym-Par (Deg) 173.2(3) 119.2(4) 5.1(4) 125.7(4)

173.2(3) 119.2(4) 5.1(4) 125.7(4)

Ring Bond Distance (Ang) 1.402(5) 1.511(5) 1.558(5) 1.521(5)

1.399(5) 1.508(5) 1.556(5) 1.507(5)

Weighted Average Ring Bond Distance = 1.4952( 18,219) Ang. - NOTE: 1st esd.

Internal, 2nd esd External.

Weighted Average Abs. Torsion Angl. = 58.35( 16,999) Deg. see: e.g.

Domenicano et al., Acta Cryst.(1975), B31, 221-234.

Cremer & Pople Puckering Parameters [D. Cremer & J.A. Pople, J.Amer.Chem.Soc.,

97, (1975), 1354-1358]

---------------------------------------------------------------------------------------

Q(2) = 0.024(4) Ang., Phi(2) = 146(9) Deg

Q(3) = 0.906(4) Ang., Phi(3) = 111.6(2) Deg

Q(4) = -0.005(4) Ang.

Total Puckering Amplitude Q = 0.906(4) Ang.

* NOTE * - A Cyclic Forward Shift of the Pivot Atom from At1 to At2 Transforms

Q(4) into - Q(4), Phi(2) into Phi(2) + 90 and

Phi(3) into Phi(3) + 135

Conformational Analysis (G.G. Evans & J.A. Boeyens, Acta Cryst. (1989), B45, 581-590)

Coefficients of Primitive and Normalised Forms

M Primitive Coefficient Angular Value

-------------------------------------------

CosForm 2 0.020 0.020 16.0

SinForm 0.013 0.013 8.0

CosForm 3 0.509 0.499 8.0

SinForm 0.472 0.463 12.0

4 0.005 -1.0

:: Note: This is a C -Form

Ring 1 Plane Angles

Least-squares planes (x,y,z in crystal coordinates) and deviations from them

(* indicates atom used to define plane)

8.0285 (0.0037) x + 4.5695 (0.0199) y - 2.3194 (0.0060) z = 7.3788

(0.0110)

* 0.0005 (0.0028) C11

* -0.0096 (0.0031) C12

* -0.0118 (0.0031) C13

* -0.0104 (0.0028) C14

* -0.0071 (0.0027) C15

* 0.0210 (0.0028) C16

* -0.0034 (0.0022) C31

* 0.0207 (0.0024) C33

Rms deviation of fitted atoms = 0.0126

1.7272 (0.0187) x + 20.0125 (0.0163) y - 2.3073 (0.0073) z = 11.3628

(0.0105)

Angle to previous plane (with approximate esd) = 59.926 ( 0.141 )

* 0.0006 (0.0020) C31

* -0.0005 (0.0019) C32

* -0.0006 (0.0020) C33

* 0.0005 (0.0019) C34

Rms deviation of fitted atoms = 0.0005

# end ring 1

Ring 2 Plane Angles

Least-squares planes (x,y,z in crystal coordinates) and deviations from them

(* indicates atom used to define plane)

7.8912 (0.0043) x + 5.7621 (0.0216) y - 2.3396 (0.0068) z = 6.6683

(0.0150)

* 0.0334 (0.0029) C21

* 0.0308 (0.0031) C22

* 0.0277 (0.0034) C23

* -0.0061 (0.0034) C24

* -0.0331 (0.0032) C25

* -0.0053 (0.0030) C26

* -0.0488 (0.0024) C32

* 0.0015 (0.0025) C34

Rms deviation of fitted atoms = 0.0282

1.7272 (0.0187) x + 20.0125 (0.0163) y - 2.3073 (0.0073) z = 11.3628

(0.0105)

Angle to previous plane (with approximate esd) = 56.732 ( 0.145 )

* 0.0006 (0.0020) C31

* -0.0005 (0.0019) C32

* -0.0006 (0.0020) C33

* 0.0005 (0.0019) C34

Rms deviation of fitted atoms = 0.0005he plane t

N1 Planarity

Least-squares planes (x,y,z in crystal coordinates) and deviations from them

(* indicates atom used to define plane)

8.0257 (0.0053) x + 4.7589 (0.0323) y - 2.2931 (0.0081) z = 7.4755

(0.0156)

* -0.0052 (0.0025) C11

* -0.0023 (0.0024) C12

* 0.0044 (0.0024) C13

* 0.0011 (0.0024) C14

* -0.0086 (0.0025) C15

* 0.0106 (0.0026) C16

Rms deviation of fitted atoms = 0.0063

8.0392 (0.0074) x + 4.5725 (0.0433) y - 2.3003 (0.0119) z = 7.4023

(0.0194)

Angle to previous plane (with approximate esd) = 0.497 ( 0.192 )

* -0.0059 (0.0010) C11

* -0.0059 (0.0011) C15

* 0.0178 (0.0032) C16

* -0.0060 (0.0011) N1

Rms deviation of fitted atoms = 0.0103

N2 Planarity

Least-squares planes (x,y,z in crystal coordinates) and deviations from them

(* indicates atom used to define plane)

7.8616 (0.0064) x + 6.2499 (0.0336) y - 2.2870 (0.0093) z = 7.0230

(0.0229)

* 0.0073 (0.0026) C21

* -0.0106 (0.0025) C22

* 0.0048 (0.0026) C23

* 0.0042 (0.0029) C24

* -0.0076 (0.0029) C25

* 0.0019 (0.0027) C26

Rms deviation of fitted atoms = 0.0067

7.9151 (0.0084) x + 6.1723 (0.0442) y - 2.2103 (0.0138) z = 7.0228

(0.0326)

Angle to previous plane (with approximate esd) = 0.806 ( 0.226 )

* 0.0017 (0.0011) C21

* 0.0017 (0.0011) C25

* -0.0051 (0.0033) C26

* 0.0017 (0.0011) N2

Rms deviation of fitted atoms = 0.0030
